# Embodying the smartwatch: How elite runners mediate data and lived corporeal experience

**DOI:** 10.1177/10126902261418739

**Published:** 2026-02-16

**Authors:** Nicolas Moreau, Bachir Sirois-Moumni, Emmanuelle Larocque, Florence Vinit, Brice Favier-Ambrosini

**Affiliations:** School of Social Work, University of Ottawa, Canada; School of Social Communication, Saint Paul University, Canada; Departement of Social Work, 59310 Université du Québec en Outaouais, Canada; Department of Psychology, 14845 Université du Québec à Montréal, Canada; Department of Science of Education, 14661 Université du Québec à Chicoutimi, Canada

**Keywords:** running, carnal sociology, data/sensation, elite runners, self-tracking, embodiement, running-related data, smartwatch

## Abstract

Given the proliferation of self-quantification technologies in running over the last few years, our research seeks to fill a significant gap by studying the relationship of elite runners with these self-tracking technologies. More specifically, this article explores the technical, corporeal, and relational dimensions of the use of smartwatches by the population of elite athletes in Quebec. Drawing on the theoretical framework of carnal sociology, and based on a qualitative methodology of 18 mobile interviews conducted during runs, our study reveals that the integration of smartwatches is a process that unfolds in two primary, chronological phases. The first phase of “becoming digital” is characterized by an initial importance attributed to data from smartwatches, where numbers take precedence over bodily sensations, relegating the sensory signals of the lived body to the background. The second phase entails a progressive reconnection to the body, marked by a continual interplay between data and sensations. Beyond this individual trajectory, our results also highlight the central role of the socializing framework. The coach and training group act as essential mediators who contextualize, embody, and at times question the data, thereby actively contributing to the co-construction of this lived data. In conclusion, our research shows that the adoption of technology by elite runners is not a purely individual process, but a socially developed skill, in which the body, the self-quantification tool, and the social environment interact to produce a more holistic form of embodied knowledge, refining and complexifying the data/sensations relationship.

## Introduction

Long-distance running is one of the most popular forms of physical activity worldwide (Hulteen et al., 2017), and its popularity has significantly risen over the past few decades, particularly among the dominant social classes (Cubizolles et al., 2017). The growing popularity of long-distance running can be linked to the social norms of late modernity, including norms of social acceleration (Rosa, 2018), performance (Ehrenberg, 2014), body ideals (Quidu and Favier-Ambrosini, 2022), and the pursuit of optimal health (Cederström and Spicer, 2016). Moreover, the advent of self-tracking technologies has profoundly transformed the contemporary running ecosystem in the Western world, reshaping the ways in which performance is measured and evaluated. This article aims to understand the sociological issues raised by self-tracking, based on the lived experiences of elite athletes.

While there is a long tradition of quantifying athletic capacities in the world of sports (Delalandre, 2009), the proliferation of data tracking devices (Dalgalarrondo, 2018) has nevertheless led to an unprecedented expansion of quantification practices. Today's athletes are adopting smartwatches, biometric bracelets, and digital applications on a massive scale, meaning these technologies have migrated from the domain of professional expertise to a space of autonomous, personal use (Quidu and Favier-Ambrosini, 2022). The use of self-tracking practices has seen a remarkable expansion since 2010, driven by the emergence of influential industry leaders such as Fitbit^®^, Apple Watch^®^, and Runkeeper^®^ (Rail, 2016). Applications like Strava^®^ take self-tracking to another level by sharing it on social media, to such an extent that this type of application now has 135 million users worldwide (Strava Press, 2025), with over 2 billion activities saved on the platform in 2024. However, Lupton (2020) and Quidu (2021) have identified a significant deceleration in this sports-leisure market dynamic, potentially correlated with the particularly high abandonment rate of wearable devices and personal health tracking technologies. Indeed, approximately one-third of American users stop using the devices within six months of acquisition (Clawson et al., 2015; Ledger and McCaffrey, 2014), a phenomenon also observable with Strava^®^, which had a 30-day retention rate of only 16% in 2021 (Alchemer, 2021). Given this empirical observation, we felt it was sociologically necessary to study the day-to-day relationship of elite runners with these self-tracking technologies and the process of datafication, “in which massive amounts of digital data are produced (…) on a continuous and real-time basis” (Mertala and Palsa, 2024: 330). This is all the more relevant given that the current scientific literature focuses mainly on recreational runners (Couture, 2021; Cubizolles et al., 2017; Quidu and Favier-Ambrosini, 2022; Toner et al., 2023).

Alongside this proliferation of technology, there is growing interest among researchers in corporeal experiences of athletic cultures (Sparkes, 2009; Toner et al., 2023; 2024), with a significant development in qualitative and phenomenological research in particular. However, Toner et al. (2023) point out that despite the promise of real-time analytics and the many studies of connected objects conducted from a variety of theoretical perspectives, few studies have focused on elite runners’ sensory and corporeal experiences of these devices. In this limited corpus on the elite runner's lived experience, Crawley's (2021) article stands out because it highlights how, unlike Western practices of individual ownership, GPS watches and digital self-tracking devices (terminology used by the author) are not objects specific to each runner, but a tool that circulates between teammates to determine the collective pace of training. Indeed, in the community of Amhara society (where Crawley runs), trainings are always collective, thus practicing a pace is mainly together as a group, with shared responsibility for the rhythm (*Ibid*).

In contrast to approaches that focus on the abandonment of these technologies (Mertala and Palsa, 2024; Quidu, 2021), our study examines how Western elite runners develop usage strategies over time that allow them to integrate these devices into their daily practice. We hypothesize that these athletes use the smartwatch as a tool for learning about and refining their perceptions rather than as a strict normative framework. In this sense, our work is in line with the perspective of Clark, Southerton and Driller (2022), who have shown the importance of moving beyond binary analyses of runners’ use of technological tools (i.e., using or not using smartwatches).

We are working in the tradition of carnal sociology (Bogue Kerr, 2024; Moreau et al., 2023a; Wacquant, 2015), which sees the body as both “subject” and “objectified,” “active” and “acted upon” (Crossley, 1995). This perspective allows us to: a) avoid the pitfalls of a strictly cognitivist approach that ignores the embodied aspect of the runner's experience; and b) move beyond the deterministic view that self-tracking necessarily leads to a degradation of lived experience in the short or long term (Etkin, 2016). We therefore hypothesize that the embodied strategies of elite athletes constitute “a bricolage of norms and possibilities, injunctions and preferences” (Dalgalarrondo and Fournier, 2019: 642) which merits study. Responding to the call from Pharabod (2019) and Dagiral et al. (2019), who stress the importance of documenting the trajectories of use of connected devices, this research therefore proposes to follow elite runners in their “process of learning to use and adopting the watch” (Quidu and Favier-Ambrosini, 2022: 528).

## Research objectives

In this article, we will explore the technical, corporeal, and relational dimensions of the use of smartwatches in the specific context of elite-level running. More specifically, our research seeks to: 1) understand the evolution of the link between corporeal expertise and digital data for the population of elite athletes in Québec (Lupton, 2020); and 2) explore the role of a third party (the coach and training group) in the construction of their “perceptive know-how” (Paintendre, Schirrer and Andrieu, 2019: 4).

## Theoretical framework

Carnal sociology aims to identify the social dimensions of lived experiences, particularly sensory or emotional ones. For Wacquant, carnal sociology “is a sociology not of the body as sociocultural object but from the body as fount of social intelligence and sociological acumen” (2015: 5). Consequently, the researcher must position themselves “not above or on the side of action but at its point of production” (Wacquant, 2015: 5). Our comprehensive, epistemological stance, is therefore to consider the social agent as a fundamentally “embodied” being (Crossley, 1995; Wacquant, 2015), which enables us to approach the actual, ordinary practices of the lived experience of self-tracking.

More precisely, the carnal sociology developed by Wacquant (2015) must be understood ontologically on two levels for our research: 1) the participants are defined as a “sensate, suffering, skilled, sedimented, and situated corporeal creature” (Wacquant, 2015: 2); 2) the researcher and the methodology used “[…] must methodically mine and thematize the fact that, like every social agent, he comes to know his object by body; and he can leverage carnal comprehension by deepening his social and symbolic insertion into the universe he studies” (Wacquant, 2015: 4). In this context, focusing on the embodied dimension of the self-tracking experience required an analytical device that put the participants and the researcher in an in-situ running context.

The theoretical framework of carnal sociology is justified for two reasons. The first is that few scientific studies have focused on the lived experience of self-tracking among elite runners. The second is that carnal sociology, while frequently used in sport sociology, has rarely been drawn upon outside ethnographic research methods.

Given that this lived experience is not innate, but part of a gradual learning process, the concepts of perceptive know-how (Paintendre, Schirrer and Andrieu, 2019) and of the capable body (Paintendre and Andrieu, 2015) will also be harnessed to highlight the fact that the use of smartwatches among elite runners is “an active, agentic, social and reflexive form of ‘work,’ often requiring mindful sense-making” (Allen-Collinson, Crust and Swann, 2018b: 1325). In this sense, it is a process of construction and learning, enabling the development of sensory expertise (Carter et al., 2022). More specifically, the concept of perceptive know-how can be understood as “an ability to listen to and interpret the sensations emanating from the body, with a view of adapting to the environment and/or better managing one's corporeal capital” (Paintendre, Schirrer and Sève, 2020: 118). It highlights the temporality of its acquisition at three levels: 1) detecting sensations; 2) interpreting them; and 3) making decisions based on them (Paintendre and Schirrer, 2024). Mapping bodies in detailed embodied ways can be achieved through the mediation of technological devices, as we will show in this study.

This concept of perceptive know-how (Paintendre, Schirrer and Andrieu, 2019) echoes intro-sensing (Kristensen and Ruckenstein, 2018), but also differs from it on several levels. First, according to the concept developed by Paintendre, Schirrer and Andrieu (2019), perceptive know-how does not necessarily occur through measuring instruments, unlike intro-sensing (Kristensen and Ruckenstein, 2018). Second, while intro-sensing is based primarily on internal sensation and its interaction with measuring instruments, the development of perceptive know-how is multifactorial, encompassing not only the physical environment, but also the social conditions (such as training alone or in a group ; relationship with the coach to discuss data) that are potentially part of sensory learning. As such, we have chosen the concept of perceptive know-how: a) because our research objectives notably aim to understand how the social runner's environment influences the perception/data relationship; b) to recognize that sensory learning is not based only on data. Importantly, our decision not to adopt the concept of data sense (Lupton, 2017) as a theoretical framework was driven by our desire to a move beyond data-focused sensory learning. Nevertheless, the concepts of intro-sensing and data sense will be used in this article, specifically to discuss our results.

The “capable” body (Paintendre and Andrieu, 2015) will be seen as the skill of perceiving what their body can “offer during movement [in our case, running] (…), an understanding of its possibilities for action” (Paintendre, Schirrer and Andrieu, 2019: 51–52).

## Methodology

### Ethical process

This research received ethical approval from the university with which the primary researcher of the study is affiliated, as well as those of several co-researchers. To preserve the anonymity of the elite runners, we have: 1) changed participants’ names; 2) carefully selected excerpts of interviews to avoid revealing their identities.

### Population and data collection techniques

Our research focuses specifically on elite runners in Québec. The status of elite runner is determined by Athlétisme Québec based on participants’ time performances over a given distance for their age and sex. This status provides financial advantages, particularly in terms of taxation, but also in terms of medical monitoring (Athlétisme Québec, n. d.). This population was specifically targeted initially for two reasons: 1) their daily (and often twice-daily) training, echoing an “ascetic way of governing the body and social life, a discipline and an ethic of effort” (Darmon, 2003: 276–277); 2) the decisive place of quantified performance in their practice.

The choice of running interviews was motivated by three primary considerations. First, from a theoretical perspective, this choice appeared consistent with the insights drawn from carnal sociology (Wacquant, 2015). Running interviews (Bogue Kerr and Moreau, 2025; Esmonde, 2019; Moreau et al., 2023a, 2023b; Palmer, 2016) allow us to understand the multisensorality, subjectivity, and spatiality of participants, in relation to their self-quantification. Several respondents, like Magalie, mentioned to us that the running interviews allowed them to “feel like [they were] reconnecting with emotions.” In this way, the proximity between the research question and the context for the interview (running in both cases) encourages the “authentic” expression of lived experiences. Secondly, the sensory, carnal, and emotional immersion of the researcher through their own body has been considered a promising avenue for gaining deeper access to the experiential and embodied knowledge of elite runners (Brown and Durrheim, 2009; Wacquant and Vanderbroek, 2024). In particular, this strengthened the rapport, trust, and even complicity with the interviewees, which contributed to the internal validity of the interviews (Bruchez, Roux and Santiago Delefosse, 2017). Thirdly, conducting the interviews during the elite athletes’ training sessions enabled us to facilitate recruitment by adapting to participants’ particularly busy schedules (Julla-Marcy et al., 2017), as indeed was mentioned by all interviewees who contributed to this study.

### Data collection

We used three strategies to recruit our participants: 1) a post on the social media pages of popular sports clubs and sports/athletics in Québec; 2) a post on the Facebook group with which the researcher carrying out the interviews is affiliated for the last 12 years; 3) the snowball sampling technique (Browne, 2005) and word of mouth. This athletic club is located in Montréal, in the province of Québec (Canada). The club operates as follows. The coach designs training plans and offers interval training sessions (usually on the track) two days a week to all of its members (elite and non-elite athletes). The training sessions are very similar for the different athletes as the difference is only in the runners’ paces. Elite athletes benefit from an additional exclusive interval session per week. The other training sessions (recovery and long run) are informal. Attendance in formal training depends on the athletes. Finally, elite athletes sometimes run alone or in groups for informal training (recovery and long run). These informal groups are very often composed of elite athletes, but can also include non-elite athletes.

Criteria for inclusion were as follows: 1) to be at least 18 years of age; 2) to speak and understand French or English; 3) to be an “elite” athlete according to Athlétisme Québec's criteria for times, age, and sex in 2022. Our definition of elite athlete status allowed us to question: 1) internationally recognized runners, while others performed mainly across Canada or Québec; 2) athletes performing over various distances, from 3000 meters (track) to the marathon.

Participants contacted the researcher by email or phone. A consent form was then sent by email for signature before the interview was carried out. Sociodemographic data was collected through a questionnaire that was completed by participants following the interview (Table 1).

**Table 1.** Sociodemographic profile of elite runner participants (n = 18).

		N	%
Gender	Man	9	50
	Woman	9	50
Average age	31.2 years old (±4.9)	18	N/A
Language	French	18	100
Civil status	Married or common-law	14	78
	Single	4	22
Ethnic background	White/European	16	89
	Black/African/Caribbean	1	5.5
	Latin-American	1	5.5
Level of education	Graduate studies completed	13	72
	Graduate studies partially completed	3	17
	College/university	2	11
Annual income^1^	Under C$50,000	4	22
	C$50,000–C$100,000	9	50
	C$100,000–C$150,000	3	17
	Over C$150,000	1	6

We conducted 18 running interviews between February and October of 2023. The duration of the interviews ranged from 38 min to 1 h and 35 min (with an average of 1 h and 2 min per interview), covered distances between 5.2 kilometers and 16 kilometers (with an average of 10.7 kilometers per interview), at a pace of 5:59 min per kilometer, in the greater Montréal region. Our semi-structured interview grid was divided into four primary themes: 1) previous athletic experiences; 2) temporal dimension of smartwatch adoption; 3) lived experience of the practice of self-tracking; 4) data use, analysis, and sharing. Individual interviews were recorded using microphones attached to the participant's and interviewer's shirts. Each microphone was connected to a recording device, placed in a running belt for the runners.

All interviews took place during recovery sessions. The running pace was discussed during initial contact between interviewer and elite athlete and, then validated at the beginning of the interview. Therefore, it was a very slow jog for elite athletes, who all mentioned the benefits of running at low intensity. Although the interviewer attempted to memorize the questions from the interview guide, there were several occasions when the interviewer referred to the guide (and read it while running) to ensure that all questions had been addressed.

Over the course of the interviews, we observed a degree of redundancy in the comments made by the elite runners, suggesting that we had reached a saturation point with regard to the objectives of our study. Although we cannot claim an absolute empirical saturation of data (Braun and Clarke, 2024), the consistency of the narratives collected and the stability of the emerging themes enable us to consider this sample to be sufficiently robust for our analysis.

### Data analysis

We carried out a vertical analysis (per interview), followed by a horizontal thematic analysis in which we compared the interviews with one another (Blanchet and Gotman, 2006). This required four steps: 1) a first reading of each interview; 2) deductive coding according to the aforementioned four themes in each interview; 3) inductive coding focused on the different layers of experience (emotion, sensation, attention, and thought) when the data did not fit into the four themes, but concerned the use of smartwatches; 4) data grouping. Thus, our theoretical framework and the concepts used, such as perspective know-how, were chosen because of their relevance following the analysis of the data. Each author of this article actively participated in the data analysis, either by coding or helping to group the data, and did so from the perspective of the “critical friend” (Smith and McGannon, 2018: 117), that is, through discussions that took place between the authors of this article. Moreover, the fact that the researcher who conducted the interviews actively participated in the coding and analysis of the data, as well as in the writing of the article, allowed us to discuss data collection through “observant participation” (Wacquant, 2010: 70), a strategy which gives access to visceral knowledge of the social and carnal lifeworlds of elite runners. The body and habitus of the researcher are fully acknowledged and can therefore be considered as a methodological tool to help convey and generate embodied sociological knowledge (Bogue Kerr and Moreau, 2025; Mears, 2013). The entire process of coding and analysis was carried out using NVivo-14 software.

### Reflexive positionning

The first author (and also the researcher who conducts elite's interview) is himself a runner within the club where many elite runners were recruited. The elite runners who participated in the research were not personally acquainted with the interviewer, although some became close friends after this research. Thus, since data collection, the first author has been training more closely with several of the elite runners interviewed. Inclusion in this new subgroup had physical and athletic repercussions for him: he lost weight and his training has intensified (7 to 8 runs per week, averaging 100 km), resulting in significantly improved performance over the past two years. The research has therefore had both social and physical consequences for the first author. Moreover, data collection may have been influenced by a shared understanding of how their common coach structures and leads training sessions. In this context, the first author may have missed questioning the elite runners on certain experiences, such as effort rating, which could have been explored with more depth because of this epistemological proximity (Gauthier and Baril, 2022). The first author owns a smartwatch and uses Strava^®^ to quantify his training. He knows how smartwatches work, which allows him to ask insightful questions specific to elite runners: how many screens do you have open on your smartwatch and why? Can you show me? Do you use the same screens throughout the year? That the first author is a proponent of self-quantification toolsis in contrast to several other authors, who take a more critical stance towards the use of smartwatches. Indeed, our research team, two of the co-authors (authors 3 and 5) of this article advocate for minimalism in sport and maintain a critical perspective on self-quantification (Favier-Ambrosini et al., 2026; Larocque, 2025). These pluralistic visions within our research team facilitated constructive exchanges as critical friends (Smith and McGannon, 2018) during data analysis and in negotiating the presentation of results. These discussions between the different authors were essential in a context where the main author, due to the evolution of his relationships with some of the elite runners interviewed : a) tended to be uncritical of elite runners; b) advocated for a noble way of using the smartwatch and therefore risk falling into a moralistic sociology.

Finally, excerpts were selected because, in addition to their relevance, they resonated with significant moments in the interview, particularly those involving physical explanations. For example, when Thomas's talks about the link between perceived effort, pace and heart rate, he mimed his heart beat with his hands and simulated his noisy breath. Thomas's narrative, accompanied by gestures and sounds was not an exception. Indeed, most of the verbal excerpts within this article were reinforced by the embodied interactions between the first author and the participants during the running interviews. In this sense, results presented in this article are directly influenced by the way we collected data.

## Findings and interpretation

We first note that all but one of the elite athletes who participated in this study use a smartwatch for self-quantification purposes. The participant who did not use a smartwatch relied on a less sophisticated watch, with basic functions including time, GPS and heart rate. Our analysis of the interviews revealed how elite runners adopted smartwatches gradually, through different socializing frameworks: the training environment, running peers, and coaches, but also the specific constraints of the practice itself, which modulate their relationship with the tool according to social contexts and accumulated experience. Our analysis also revealed two phases in the process of engaging with lived data (Allen-Collinson and Owton, 2015; Hockey, 2013): 1) a predominant focus on digital data, relegating the lived body (Andrieu, 2011) to the background; 2) the development of perceptive know-how (Paintendre, Schirrer and Andrieu, 2019) through an ongoing and complex interplay between data and sensations (Lupton, 2017, 2020). Finally, we highlight the essential roles of the coach and the training group in this critical contextualization of data for elite runners.

### Phase 1: Becoming digital: Initiating the body to self-quantification

Our study finds that elite runners either incorporate the smartwatch through self-interest or are introduced to tracking devices by their coach. One element that stands out is that the entry into the world of smartwatches is initially characterized by an almost compulsive fascination with the smartwatch and a strong dependence on the data: “It changed my life when I got my smartwatch and it would tell me my time and pace per kilometer. It increased my motivation (…), it really changed everything, and from then on, I couldn’t live without my [smart]watch” (Camille). This period is marked by use of the smartwatch, sometimes driven by the imitation of peers, and focused on the organization of structured sessions:

We were starting to do more structured intervals and I saw that people had GPS [Global Positioning System] watches that were able to measure their distance, and I really saw it as a tool, as an advantage to use that in training. It was really at that moment, when I saw other teammates with them, that I thought it would be beneficial for me. It wasn’t necessarily recommended by the coach. The coach ran with us mostly for our warm-ups and cool-downs, and he had one himself, because he wanted to measure the distance (…) everyone had one, and I saw the benefits of having one, because I could see the pace I was running at, the distance. It made it more serious, more structured, more competitive to have a watch (Thomas).

While Thomas describes a social environment gradually leading him to adopt the smartwatch, other participants recount a more guided introduction through coaching. When the tracking devices are incorporated through prescriptive coaching practices, some participants report a more paradoxical experience, where the smartwatch does not match the body's needs or self-perceived capacities: “my coach used to make me do a lot of [30 s slower than marathon pace]. It's really hard for me to know if I’m getting there. I can’t do it because […] there's no race where I’m going to do that [run at that pace]” (Patrice). Myriam said: “It was for intervals, [the coach] was saying 5 min 30 [per] kilometer. So, I just went with the flow”. The “prescribed” self-tracking integration period can therefore involve a certain level of discrepancy between how the coach configures the smartwatch and the athlete's perceived bodily capacities and efforts required to train according to them.

Motivations for adopting the smartwatch varied according to the specific context of each runner. A new environment, the need to train alone, a provincial selection, athletic evolution, and aesthetics were all mentioned as reasons for choosing a smartwatch.

For some, deciding to start using a smartwatch was rooted in a change of environment: “25 kilometers, I don’t really know what that looks like for me when you’re running in Montréal (…) it became a little more important when I arrived here 7 years ago, for training” (Romain). For others, like Elodie who is accustomed to shorter race distance [3000 Meters], the transition to long distance represented a change in the training “environment” itself and a sensory challenge. She explained: “I look at my pace, and I have a bit of a hard time knowing, long distance on the road is so new, that for example if someone tells me: ‘OK, we’re doing 25 kilometers at 4 min and 35 s [per kilometer]’; I don’t know what 4 min and 35 s is, this feeling is totally new (…) you tell me 3 min 10 s per kilometer, perfect, I’ll do it.” Contrasting her experiences between the familiar fast pace (3:10/km) and the new endurance pace (4:35/km), she seems aware of her lack of proprioceptive knowledge making the smartwatch a tool for learning and calibration of effort. Similarly, Patrice spoke of the difficulties of tuning in to the body to respect a certain pace. In this context, the quantification device serves as the pace-keeper for his solo runs: “I started to train on my own a bit more, and also needing more to see if I was keeping to the pace.” For Sophie, the switch to a more sophisticated smartwatch was explained by a provincial selection:

2015 (…) It was the first time I was selected for the Québec cross-country team. I think I had a Garmin^®^ 220, the first model. (…) I realized that I wanted to have time reference points, not just on the track but everywhere I went. No matter what route I was taking. So that's when the [smart]watch is going to help me get that kind of data.

The transition to a more sophisticated smartwatch can also be part of an athletic evolution:

After four or five events, I realized that everyone had [smart]watches, (…) and then I realized that I was always starting out too fast, sometimes the end of races was tough, people were catching up with me, or else, I didn’t really know how to know I was [running] at what speed (…) I didn’t even understand what the pace was, so at some point, I thought: ‘OK, I think I need a [smart]watch.’ So I went and bought a Garmin^®^ watch (Mathias).

This initial phase of integration reveals a learning process in which elite runners learned to decipher and use this new information as primary reference points for effort in a context in which sensory experience remains minimal: “it's hard to know your level of effort (…) when you start [the race]” (Patrice). Gaining self-quantification literacy and adapting to this external device involves, during this first phase, a certain level of separation from the bodily sensations of running, as suggested by Xavier: “[the [smartwatch]] was my reference (….) otherwise, I didn’t know what pace I was going at (…), I didn’t have running experience yet (…) I didn’t know the feeling yet.” Data becomes the main indicator of how fast to run and how far to go. The process of “becoming digital” therefore involves a focalized attention on the smartwatch, the number (and not the lived body) being at the centre of the pace to adopt. However, after this first phase, the runner learns to (re)connect to the body with more assurance and confidence, as suggests Patrice: “I really learned how to train according to effort (…) 15-kilometer effort, 10-kilometer effort, 5-kilometer effort.” It is only after this first phase, where the attention of elite runners is focused on data, that their perceptive know-how (Paintendre, Schirrer and Andrieu, 2019) can begin to develop.

### Phase 2: The development of perceptive know-how through progressively reconnecting to the body

Following the initial transition of “becoming digital” where the focus is mainly on the self-tracking device, data analysis reveals a progressive reconnection to the body and senses. More specifically, elite runners develop, through the accumulation of timed experiences, “perceptive know-how,” in other words, an “ability to use their sensorality to act and make decisions when running” (Paintendre, Schirrer and Andrieu, 2019: 4). This process enables elite runners to develop a dual expertise in their bodies: numerical and sensory and thus facilites the development of the athlete's “capable body” (Paintendre and Andrieu, 2015). The development of this sensorial expertise through technological instruments such as the smartwatch directly echoes the concept of intro-sensing (Kristensen and Ruckenstein, 2018), as illustrated by Thomas who uses the data from his [smart]watch to hone his reading and understanding of his heart rate: “I can [run] at 4 min and 30 s per kilometer. But does my pulse really reflect an effort of 4 min and 30 s per kilometer? (…) I only recently started to (…) track and look more consciously at (…) my heart rate”. Similarly, Sarah suggested: “when it comes to heart rate (…), I feel it when my heart is racing or not (…) I look at my [smart]watch, I see that I’m not going slow enough”. Using the smartwatch to slow the pace was also observed among Ethiopian elite runners (Crawley, 2021), suggesting that this specific action in relation to the smartwatch is important for elite runners.

This perceptive know-how is refined over time through the repetition of similar efforts (Camille: “I’ve done a lot of 5 Ks in my time, I know how I’m supposed to feel”), allowing runners to develop their capable body (Paintendre and Andrieu, 2015), as Romain also suggested: “with laps on a track, you start to know your paces by heart, and then over 400 meters, you basically know all your lap times and you know how fast you’re running.”

While it is learned over time, the development of this sensorial expertise can also be acquired over the course of a single training session: “for long [runs], when it lasted 1 h and 40 min, the first 5 kilometers, I used the [smart]watch a lot as a reference, and then I was fine after that” (Camille). Just as studies have shown that it is fruitless to analyze the relationship with smartwatches in a binary way (Clark, Southerton and Driller, 2022), the watch is also not used in the same way throughout the course of a single training session (Esmonde, 2020; Quidu and Favier-Ambrosini, 2022).

This newly constructed capable body (Paintendre and Andrieu, 2015) deepens on the complexity of knowledge available to runners, enabling them to adapt their training sessions to the best of their ability by taking into account both the digital data and the way they feel that day, as Paintendre, Schirrer and Andrieu (2019) were able to observe in the learning of step fitness in physical education. Three main processes therefore caracterize this phase 2 and the development of perceptive know-how (Paintendre, Schirrer and Andrieu, 2019) and the fabrication of lived data (Lupton, 2017, 2020): 1) establishing a system of equivalence between quantitative data and sensory dimensions; 2) using the smartwatch as a tool for retrospective validation; 3) managing the lack of consistency between sensations and quantitative data. These three processes directly echo those mentioned in the theoretical framework: 1) detecting sensations; 2) interpreting them; and 3) making decisions based on them (Paintendre and Schirrer, 2024).

The first way shows how some runners develop a hybrid evaluation system. The most striking example is the use of a perception scale combining objective data and sensations that is refined over time. Elite runners are therefore actively participating in “data sense-making” (Lupton, 2017, 1604), which involves making decisions about the value of data and how it can be incorporated into their athletic practice: “[In the context of a training session] you think: ‘OK, I must be at 8, I must be at 7’[this number represents a simple self quantification strategy of the athletes rating its effort on a scale of 1 to 10], then, I’m nearly at the top and I’m much further than at my planned speed. If your effort level doesn’t match your speed, you use your watch to find out what's going on. How do you react? How do you adapt?” (Julie). Adaptation is at the heart of “perceptive know-how” (Paintendre, Schirrer and Andrieu, 2019). As the following excerpt illustrates, the elite athlete's bodily experience is linked to the environmental context. Thus, the data are analyzed depending on the season (winter) and the elevation gain: “during the pandemic, I trained a lot on my own and in the winter I did a lot of hills (…) my intervals, when I changed them, I did them uphill but now with the stride, I could work more on power rather than on speed (…) if I want to work at 3 min 20 uphill [pace km/h], it's clear that I'm not going to do 3 [minutes], 3 [minutes], 20 [secondes] (…) I'm going to [find a equivalence to be] at 285 watts on the hill “(Sophie). It is in this active mediation between the data (the number), the body (the sensation) and the environment (the terrain) that the runner's perceptive know-how is expressed. Here, the runner is actively interpreting the numbers to make sense of them. The data is no longer considered as a fixed, autonomous, and self-sufficient entity, but rather as an element that is actively integrated into the athlete's lived experience.

The second way results from the use of data from the smartwatch for retrospective validation rather than as a guide in real time. This can be done after daily training (Xavier) or after several weeks (Alexandre). As such, Xavier stated: “[I] look at my paces (…), the distance covered above all, the number of steps, but also the heart rates” after each run. Alexandre said he will “use [the smartwatch] after training mostly or try to see if there is an evolution over a cycle of five or six weeks of the same training.” Therefore, developing perceptive know-how (Paintendre, Schirrer and Andrieu, 2019) requires some reflexive work (Allen-Collinson, Crust and Swann, 2018b) on the part of the elite runner over a shorter or longer period of time. This reflexive work between the data objectivized by the watch and corporeal sensations, implies an active engagement in a process of interpretation that shapes their understanding of their own performance.

Managing the lack of consistency between daily sensations and the data from the smartwatch can be put into dialogue with the concept of “disrupted embodiment” (Toner et al., 2023: 812), where in the case of fatigue, injury, or difficult environmental conditions, runners report a “disruption” of the relationship between data and sensations (Lupton, 2020).

Elite runners seem to use two main strategies, which are not mutually exclusive, to manage this discordance between digital data and sensations. The first is to put the smartwatch data into perspective (Fresco and Espiritu, 2025) and establish a clear hierarchy, to give priority to the capable body (Paintendre and Andrieu, 2015), as illustrated by comments from Camille (“ultimately, it's my body that's going to tell me what to do”), and Antoine (“there are days when I feel good, and days when I don’t feel good, and if I go by the watch, that means that the watch is smarter than my body, and I’ve had doubts about that since early in my career”), as well as Mathias, who suggested that “the watch will never tell you how you’re feeling that day.”

The second strategy is to contextualize the information provided by the smartwatch. Elite athletes, like amateur athletes (Quidu and Favier-Ambrosini, 2022), use external attributes (environmental conditions, work) to explain this “disrupted embodiment” (Toner et al., 2023). As Alexandre said: “I had a long day at work […] maybe I spent more energy on this other thing today.”

Runners’ ability to contextualize their underperformance can be interpreted as an illustration of “situated objectivity” (Pantzar and Ruckenstein, 2017), in which digital data, far from being perceived as raw or universal truths, becomes interpretive material for refining the capable body (Paintendre and Andrieu, 2015), which allows elite runners to “adjust expectations of what is achievable during specific sessions” (Toner et al., 2023: 812), as illustrated by Paul:

I look at my watch, I look at the pace a bit, I look at the times, I go: ‘Aye, we’re not going to get the time we wanted today.’ So you keep up the effort, then I don’t know, I slowed down because it wasn’t going well, but I didn’t slow down on purpose. I just kept giving my best, but that was my best for that day.

While the scientific literature has shown us the importance of experience in the construction of this “lived data” (Allen-Collinson and Owton, 2015; Hockey, 2013), we will see that the coach and the group are also essential for the development of perceptive know-how (Paintendre, Schirrer and Andrieu, 2019) among elite runners.

### The production of lived data by the coach and the group

Self-tracking should be understood not as a single relationship between the individual and their technology device, but as a communication process, as Lomborg and Frandsen (2016) have shown with regard to the importance of peers, particularly in terms of motivation. In this context, somatic learning (Shilling, 2017) is not developed solely through individual work. Rather, it is shaped by the athletic culture in which the athlete is immersed, as shown by Allen-Collinson et al. (2018a) with their research on the importance of socialization and learning in the experience of temperature among athletes. More specifically, the coach and training group play an essential role in the development of this sensory expertise. Firstly, the coach produces data through the training plans the elite athlete must follow: “He [the coach] would hang a chart on the wall, and then you would check your name and it would say the speed for the day” (Myriam). Secondly, the coach vocally embodies the data they have produced by giving it a voice, a physical presence: “I listen more to [what] [my coach] is going to tell us about our times around the 200 for example or 400 meters […] versus looking at my watch” (Claire). Thirdly, the coach, after having developed and embodied the numbers, will put them in perspective by highlighting the different variables that explain a given speed: “[my coach] talked to me a lot about the level of effort, about managing your effort based on how you feel, and not always according to data that might vary over time” (Mathias). This contextualization and analysis of data by the coach was illustrated by Rapp and Tirabeni (2018) who also highlighted the importance of non-hierarchical discussions around data and sensation between the coach and elite athletes to calibrate their training.

This form of social mediation of data by the coach echoes the work of Borenstein regarding Ethiopian women runners (2021), though with distinct motivations. Borenstein (2021) describes how the smartwatch becomes a “collective instrument” (Borenstein, 2021: 9) and “incorporate(s) a network of people and entities” (Borenstein, 2021: 15). In both our context and theirs, the device is rarely a solitary tool. However, where our participants sometimes engage in a pedagogical dialogue with the coaches to interpret and refine data and sensation, Borenstein (2021) shows how the runners navigate a relationship where the data is monitored, extracted and managed by others, be it coaches (who are also sometimes husbands), agents or scientists to ensure productivity and forms of surveillance. While some of our interviewees had partners who were also running-mates, and who sometimes introduced them to the smartwatch, these dimensions weren’t observed in our research.

The training group is the second technique for refining this data/sensations relationship (Lupton, 2020). By following the group's pace, elite athletes sometimes run at speeds that don’t correspond to the times predicted by the numerical data: “[when we do] intervals, I’d say that's maybe the time I look at my watch the least, and I go more by (…) the group” (Camille). Myriam continued: “I was running with a guy and a girl, we were always together (…), we always tried to follow whoever was in the best shape on that particular day.” The fact of running in a group and at sometimes unusual speeds doesn’t mean they forget their smartwatch, which remains a constant tool for adjustment: “in training sessions, sometimes I’m with the group in the front, but I can see that my heart rate is too high, so I consciously slow down” (Thomas). Here, the group allows runners to develop new perceptive know-how (Paintendre, Schirrer and Andrieu, 2019) by running at speeds not predicted by digital data from the training plan.

The smartwatch then quantifies these new sensations, making the elite runner's capable body more complex (Paintendre and Andrieu, 2015). A kind of dialectic seems to arise between the discontinuity of the different rhythms brought about by the presence of the group and the stability of the individual reference point of the smartwatch. This takes us straight back to the studies that have shown the importance of others in the development of sensorality, as Allen-Collinson, Blackwell and Henderson (2025) have done for outdoor activities, or McNarry, Allen-Collinson and Evans (2021) with the concept of “somatic culture” among swimmers.

## Discussion

Our research highlights that the self-quantified digital body is indeed a “point of production” of knowledge (Wacquant, 2015: 246). The ascetic practice (Darmon, 2003) of running, paired with a secondary socializing framework (coach and training group), allow elite runners to develop in-depth knowledge of their perceptive know-how (Paintendre, Schirrer and Andrieu, 2019). The guidance of a coach enables an “assisted” use of the smartwatch, which in turn, encourages a reflective and more conscious use of self-tracking devices. This dual expertise—corporeal and interpretive—confers a privileged position for considering the sociotechnical and “lived informatics” (Rooksby et al., 2014) issues related to the use of the smartwatch in athletic practice, in a context where elite athletes integrate self-quantification devices with the intention of becoming more sensitive to the relationship between numerical data and corporeal sensations.

More specifically, our results show the existence of two phases in the adoption of smartwatches among elite runners. In the first phase, our participants focus primarily on the digital data, relegating lived body data (Andrieu, 2011) to the background with the aim of mastering the smartwatch. The constant back-and-forth between data and sensations (Fresco and Espiritu, 2025; Lupton, 2017, 2020; Kristensen and Ruckenstein, 2018) occurs in the second phase, which allows elite runners to develop and refine their perceptive know-how (Paintendre, Schirrer and Andrieu, 2019). Participants use limited smartwatch data (GPS, time) to develop sensorial expertise, which is also what Borenstein (2021) found in her study of Ethiopian female elite athletes. However, “self-tracking” in Borenstein's analysis collapsed into “tracking devices” for the network of agents, sponsors and spouses surrounding the athletes. This data extracted value from the athlete's labor, extending the primary function of the device to one of accumulation and surveillance rather than the somatic attunement we observed. Thus, while the coach and training group help refine this embodied knowledge in both contexts, the socioeconomic ends of this knowledge production differ significantly in our context.

The relationship between the tracking device, the body of the elite athlete, and the coach or group, therefore allows for a fine-tuning of their internal and external “somatic sensing” capacities (Tholander, 2025: 1). Unpacking this relationship and embodied interaction contributes to deepening our understanding of felt experiences of technology and the body in sports, where a mediator (a coach or group) can, in fact, influence the runner's experience, perceptive know-how (Paintendre, Schirrer and Andrieu, 2019), and “somatic mode of attention” (Csordas, 2002).

Moreover, as has been demonstrated in other studies of self-tracking technologies, namely William'’s (2015) study on weight-loss technologies, this research highlights the sometimes paradoxical or contradictory felt experiences that can arise during the novel stages of this relationship. This helps to further understand the types of negotiations and meaning-making practices that elite runners go through with the self-tracking device and their bodily sensations. In this perspective, the coach or the training group contributes to this fine-tuning and attunement to both bodily sensations and digital information during the integration process.

The sociological framework, inspired by carnal sociology, enabled us to explore the world of self-tracking in running among elite athletes. From a methodological point of view, while Wacquant (2015) favours ethnography as a means of accessing bodily experience, our results show the heuristic value of running interviews (Bogue Kerr, 2024; Moreau et al, 2023a; Moreau et al., 2023b; Quidu et al., 2025) for capturing runners’ embodied experience. In this sense, as mentioned by Quidu (2022), carnal sociology is not the exclusive preserve of a single method.

### Limitations

As we mentioned in the methodology section, given that we are runners, our position could be described as observational participation in the sense that one of the researchers is “part of the milieu” (Soulé, 2007: 134) of the runners, since the interviewer shares the same coach as several of the elite runners interviewed and trains with them on a daily basis (while not belonging to the “elite” category according to the criteria of Athlétisme Québec).

However, we don’t think that the mobile interviews caused any significant disruptions, even if our presence and our questions did not allow the elite runners to freely express what they were feeling in the moment, in contrast to self-explanation interviews (Quidu and Favier-Ambrosini, 2022), which could have been another possible data collection method in this context. This technical issue has to be taken in consideration, particularly with regard to the flow of discussion, which was sometimes interrupted.

Lastly, while our sample was evenly divided between men and women (n = 9 for each group), this research has certain limitations in terms of a lack of analysis of the relationship between bodies, power, and the construction of identities, particularly with regard to experiences differentiated by gender or race (Stoler, 2022). While Borenstein (2021) elucidated how data is used to control and monitor the labor of Ethiopian women athletes at both the micro-level (coaches/husbands) and macro level (Global North sponsors/agents/researchers), evidence of such dynamics remained limited in the present project. Our thematic analysis did not reveal gender-specific results despite the parity of our sample and dimensions of surveillance such as weight control enabled by GPS were not explicitly mentioned by the participants. Furthermore, ethnic background was not taken into consideration due to the low number of elite runners who identified as part of a minority group (n = 2). Within the context and scope of this present analysis, the extractive dynamics and control enabled by GPS watches and the network surrounding the athlete were not observed or mentioned by our participants.

## Concluding thoughts

Our research has demonstrated, as Rapp and Tirabeni (2018: 19) mention, “understanding the body is an essential competence that is worth learning”. More specifically, we have highlighted the importance of a socializing framework in the development of the perceptive know-how (Paintendre, Schirrer and Andrieu, 2019) of elite runners, which is therefore not solely the result of their accumulated experience. Indeed, the process of refining and enhancing sensorial expertise (Paintendre, Schirrer and Andrieu, 2019) also requires secondary socialization (Darmon, 2016), carried out mainly by the coach and the training group. This key finding is especially significant as it contributes to a better understanding of the inter-relationships between the elite runner's embodied experience of running, the external feedback of the digital data and of the coach or group which participate in a specific sporting culture. It is this “culture” of distance running that contributes to athletes’ knowledge on how to construct a particular form of perceptual expertise, built upon constant interaction between body's sensation, digital feedback from the smartwatch and most importantly, the social environment in which this collective mediation takes place (Allen-Collinson et al., 2018a; McNarry, Allen-Collinson and Evans, 2021). This relationship is a novel angle that merits further investigation in sport sociology.

While this research is one of the rare studies of the experiential and technological universe of elite athletes, it remains exploratory and would benefit from further research. We believe, for example, that it would be relevant to further explore the comparison techniques (like training diaries) used by elite runners to refine the data/sensations relationship (Lupton, 2017, 2020), the construction of perceptive know-how (Paintendre, Schirrer and Andrieu, 2019), as well as the dialogue with the coach (Rapp and Tirabeni, 2018). None of elite athletes of our sample are smartwatchless, even though their use depending on the runner, the time of season, and their health status (injury). In this context, it would be interesting to study the relationship to sensations of elite runners who refuse to have a smartwatch. Indeed, studies on minimalism (of which the refusal to wear a smartwatch is a characteristic) have focused largely on amateur runners and trail runners (Quidu et al., 2025), but few on elite runners.

## Notes

1. Two participants preferred to not respond to this question.

## References

[bibr1-10126902261418739] Alchemer (2021, March 30) Strava’s missed opportunity to drive mobile app retention and gain actionable product feedback. In: Alchemer Blog. Available at: https://www.alchemer.com/resources/blog/tough-love-tuesday-stravas-missed-opportunity-to-drive-mobile-app-retention-and-gain-actionable-product-feedback/.

[bibr2-10126902261418739] Allen-Collinson J Blackwell J Henderson H (2025) Sensory pleasures and displeasures of the outdoors: Somatic learning and the senses. The Senses and Society 20(1): 34–48.

[bibr3-10126902261418739] Allen-Collinson J Crust L Swann C (2018b) Endurance work’: Embodiment and the mind-body nexus in the physical culture of high-altitude mountaineering. Sociology 52(6): 1324–1341.

[bibr4-10126902261418739] Allen-Collinson J Owton H (2015) Intense embodiment: Senses of heat in women’s running and boxing. Body & Society 21(2): 245–268.

[bibr5-10126902261418739] Allen-Collinson J Vaittinen A Jennings G , et al. (2018a) Exploring lived heat, “temperature work,” and embodiment: Novel auto/ethnographic insights from physical cultures. Journal of Contemporary Ethnography 47(3): 283–305.

[bibr6-10126902261418739] Andrieu B (2011) Sentir sa santé. In: Klein A (ed) Les Sensations de Santé: Pour une épistémologie des Pratiques Corporelles du Sujet de Santé. Nancy: Presses de l'Université de Nancy, 259–282.

[bibr300-10126902261418739] Blanchet A and Gotman A (2006) L'entretien. Armand Colin.

[bibr7-10126902261418739] Athlétisme Québec (n. d.) *Haut niveau*. Available at: https://www.athletisme-quebec.ca/athlete/haut-niveau/ (accessed June 19, 2025).

[bibr8-10126902261418739] Bogue Kerr S (2024) Research on the run: A carnal sociological approach to running interviews. Qualitative Research in Sport, Exercise and Health 17(2): 108–122.

[bibr9-10126902261418739] Bogue Kerr S and Moreau N (2025) Running and stumbling to recovery: A carnal sociological study of change in substance use. Sociology of Health and Illness 47(5): 1–12. DOI: 10.1111/1467-9566.70052. PMC12124470 40445864

[bibr10-10126902261418739] Borenstein H (2021) Tracking work from the wrist: The surveillance of Ethiopian women athletes for capital. Catalyst : Feminism, Theory, Technoscience 7(1): 1–20.

[bibr11-10126902261418739] Braun V Clarke V (2024) Thematic analysis. In: Michalos AC (ed) Encyclopedia of Quality of Life and Well-Being Research. Dordrecht, Heidelberg, New York, London: Springer International Publishing, 7187–7193.

[bibr12-10126902261418739] Brown L Durrheim K (2009) Different kinds of knowing: Generating qualitative data through mobile interviewing. Qualitative Inquiry 15(5): 911–930.

[bibr13-10126902261418739] Browne K (2005) Snowball sampling: Using social networks to research non-heterosexual women. International Journal of Social Research Methodology 8(1): 47–60.

[bibr14-10126902261418739] Bruchez C Roux P Santiago Delefosse M (2017) Validité et qualité en recherche qualitative: Aspects pratiques. In: Santiago Delefosse M Del Rio Carral M (eds) Les Méthodes Qualitatives en Psychologie et Sciences Humaines de la Santé. Paris: Dunod, 225–241. DOI: 10.3917/dunod.santi.2017.01.0225.

[bibr15-10126902261418739] Carter TF Heath S Jacobs S , et al. (2022) Sensory ecologies: The refinement of movement and the senses in sport. The Senses and Society 17(3): 241–251.

[bibr16-10126902261418739] Cederström C Spicer A (2016) Le syndrôme du bien-être. Paris: Éditions l’Échappée.

[bibr17-10126902261418739] Clark M Southerton C Driller M (2022) Digital self-tracking, habits and the myth of discontinuance: It doesn’t just ‘stop’. New Media & Society 26(4): 2168–2188.

[bibr18-10126902261418739] Clawson J Pater J Miller A , et al. (2015) No longer wearing: Investigating the abandonment of personal health-tracking technologies on Craigslist. In: Proceedings of the 2015 ACM International Joint Conference on Pervasive and Ubiquitous Computing, 647–658.

[bibr19-10126902261418739] Couture J (2021) Reflections from the ‘strava-sphere’: Kudos, community, and (self-) surveillance on a social network for athletes. Qualitative Research in Sport, Exercise and Health 13(1): 184–200.

[bibr20-10126902261418739] Crawley M (2021) Tracking selves or tracking relationships? Means of measuring time amongst Ethiopian runners. Journal of the Royal Anthropological Institute 27(3): 653–671.

[bibr21-10126902261418739] Crossley N (1995) Merleau-Ponty, the elusive body and carnal sociology. Body & Society 1(1): 43–63.

[bibr22-10126902261418739] Csordas TJ (2002) Body, Meaning, Healing. New-York, NY: Palgrave Macmillan.

[bibr23-10126902261418739] Cubizolles S Baron B Lacroix E (2017) S’engager dans les courses à pied d’endurance: Trente ans de travaux de sociologie du sport en France. Loisir et Société / Society and Leisure 41(3): 333–350.

[bibr24-10126902261418739] Dagiral É Dessajan S Legon T , et al. (2019) Making room for numbers in self-care: A sociology of self-tracking and quantification practices at different stages of life. Réseaux 216(4): 119–156.

[bibr25-10126902261418739] Dalgalarrondo S (2018) Surveiller et guérir le corps optimal: Big data et performance sportive. L'Homme & la Société 207(2): 99–116.

[bibr26-10126902261418739] Dalgalarrondo S Fournier T (2019) Introduction: Les morales de l’optimisation ou les routes du soi. Ethnologie Française 49(4): 639–651.

[bibr27-10126902261418739] Darmon M (2003) Devenir anorexique. une approche sociologique. Paris: La Découverte.

[bibr28-10126902261418739] Darmon M (2016) La socialisation. Paris: Armand Colin.

[bibr29-10126902261418739] Delalandre M , 2009. Sociologie des sciences de la performance sportive en France . Unpublished PhD Thesis. Université Paris-Est, France.

[bibr30-10126902261418739] Ehrenberg A (2014) Le culte de la performance. Paris: Calmann-Lévy.

[bibr301-10126902261418739] Esmonde K (2019) Training, tracking, and traversing: digital materiality and the production of bodies and/in space in runners' fitness tracking practices. *Leisure Studies* 38(6): 804-817. DOI: 10.1080/02614367.2019.1661506.

[bibr31-10126902261418739] Esmonde K (2020) There’s only so much data you can handle in your life’: Accommodating and resisting self-surveillance in women’s running and fitness tracking practices. Qualitative Research in Sport, Exercise and Health 12(1): 76–90.

[bibr32-10126902261418739] Etkin J (2016) The hidden cost of personal quantification. Journal of Consumer Research 42(6): 967–984.

[bibr33-10126902261418739] Favier-Ambrosini B Linossier Y Quidu M (2026) Courir pieds nus pour se reconnecter sensoriellement à la nature? Autoethnographie collective d’une transition minimaliste. Recherches & éducations 30-31: 1–16. DOI: 10.4000/14l9j.

[bibr34-10126902261418739] Fresco E Espiritu JE (2025) The watch is wrong today’: Older Canadians’ technologically mediated experiences of running. International Review for the Sociology of Sport: 1–18. DOI: 10.1177/10126902251358504.

[bibr35-10126902261418739] Gauthier M-C Baril A (2022) La proximité épistémique comme concept clé pour penser la notion d’allié.e dans les recherches en travail social auprès des groupes marginalisés. Nouvelles Pratiques Sociales 33(1): 134–153.

[bibr36-10126902261418739] Hockey J (2013) Knowing the ‘going’: The sensory evaluation of distance running. Qualitative Research in Sport, Exercise and Health 5(1): 127–141.

[bibr37-10126902261418739] Hulteen RM Smith JJ Morgan PJ , et al. (2017) Global participation in sport and leisure-time physical activities: A systematic review and meta-analysis. Preventive Medicine 95: 14–25. 10.1016/j.ypmed.2016.11.027 27939265

[bibr38-10126902261418739] Julla-Marcy M Burlot F Le Mancq F (2017) Socialisations temporelles dans le sport de haut niveau: De la maîtrise du chronomètre à la maîtrise du temps. Temporalités 25. 10.4000/temporalites.3713.

[bibr39-10126902261418739] Kristensen DB Ruckenstein M (2018) Co-evolving with self-tracking technologies. New Media & Society 20(10): 3624–3640.

[bibr302-10126902261418739] Larocque E (2025) Ecological grief and embodied place-attachment processes in nature-based sport: unsettling dominant body/land dynamics through cosmic attunement. *Sport, Ethics and Philosophy*: 1-21. DOI: 10.1080/17511321.2025.2609582.

[bibr40-10126902261418739] Ledger D McCaffrey D (2014) Inside Wearables: How the Science of Human Behavior Change Offers the Secret to Long-Term Engagement. Cambridge, MA: Endeavour Partners.

[bibr41-10126902261418739] Lomborg S Frandsen K (2016) Self-tracking as communication. Information, Communication & Society 19(7): 1015–1027.

[bibr42-10126902261418739] Lupton D (2017) Feeling your data: Touch and making sense of personal digital data. New Media & Society 19(10): 1599–1614.

[bibr43-10126902261418739] Lupton D (2020) Wearable devices: Sociotechnical imaginaries and agential capacities. In: Pedersen I Iliadis A (eds) Embodied Technology. Cambridge, MA: MIT Press, 48–69.

[bibr44-10126902261418739] McNarry G Allen-Collinson J Evans AB (2021) Sensory sociological phenomenology, somatic learning and ‘lived’ temperature in competitive pool swimming. The Sociological Review 69(1): 206–222.

[bibr45-10126902261418739] Mears A (2013) Ethnography as precarious work. The Sociological Quarterly 54(1): 20–34.

[bibr46-10126902261418739] Mertala P Palsa L (2024) Running free: Recreational runners’ reasons for non-use of digital sports technology. Sport in Society 27(3): 329–345.

[bibr47-10126902261418739] Moreau N Larocque E Jaimes A Vinit F Quidu M and Favier-Ambrosini B (2023a) Embodying or resisting social normativity? A carnal inquiry into exercise addiction experiences. Social Science & Medicine 327: 1–8. DOI: 10.1016/j.socscimed.2023.115948. 37216825

[bibr48-10126902261418739] Moreau N Quidu M and Larocque E (2023b) On becoming an “exercise addict”: Analyzing exercise addiction processes through the sociology of dispositional and contextual plurality. STAPS. Revue internationale des sciences du sport et de l’éducation physique 143(5): 99–121. DOI: 10.3917/sta.143.0099.

[bibr49-10126902261418739] Paintendre A Andrieu B (2015) Le corps capacitaire des adolescent(e)s : Une émersion du vivant dans leur perception du step. Stap 108(2): 49–59.

[bibr50-10126902261418739] Paintendre A Schirrer M (2024) Enseignement du yoga et apprentissages sensoriels : Une éducation au bien-être corporel par la dialectique confort-incofort postural ? Recherches & éducations 27: 1–16.

[bibr51-10126902261418739] Paintendre A Schirrer M Andrieu B (2019) Développer des savoir-faire perceptifs en Éducation physique et sportive: Analyse de l’activité d’élèves engagés dans une séquence d’enseignement de step. Activités 16(1): 1–29.

[bibr52-10126902261418739] Paintendre A Schirrer M Sève C (2020) Pour une éducation sensorielle à travers les activités physiques: Analyse de pratique et pistes professionnelles. *eJRIEPS* (Special issue 3). 10.4000/ejrieps.4382.

[bibr53-10126902261418739] Palmer C (2016) Research on the run: Moving methods and the charity ‘thon.’. Qualitative Research in Sport, Exercise and Health 8(3): 225–236.

[bibr303-10126902261418739] Pantzar M and Ruckenstein M (2017) Living the metrics: Self-tracking and situated objectivity. *Digital Health* 3: 1-10. DOI: 10.1177/2055207617712590. 10.1177/2055207617712590 PMC6001216 29942604

[bibr304-10126902261418739] Pharabod AS (2019) Faire ses 10 000 pas », vraiment ? Une enquête sur les pratiques de self-tracking ordinaires. *Réseaux* 4(216): 157-187.

[bibr54-10126902261418739] Quidu M (2021) L’auto-quantification de son activité sportive altère-t-elle la qualité de l’expérience vécue? Un scénario possible de l’abandon massif des pratiques de self-tracking. Implications Philosophiques. https://www.implications-philosophiques.org/lauto-quantification-de-son-activite-sportive-altere-t-elle-la-qualite-de-lexperience-vecue .

[bibr55-10126902261418739] Quidu M (2022) La participation observante de loïc wacquant comme nouvelle doxa méthodologique dans les recherches en sciences sociales sur les sports de combat? Arguments pour la réhabilitation d’un pluralisme réflexif et critique. Loisir et Société / Society and Leisure 45(2): 218–242.

[bibr56-10126902261418739] Quidu M and Favier-Ambrosini B (2022) Quelles expériences intimes et pratiques effectives de la course à pied quantifiée? Étude des usages « ordinaires » des montres connectées chez des coureurs et coureuses amateur.es à partir d’une auto-explicitation en temps réel. Loisir et Société / Society and Leisure 45(3): 506–549. DOI: 10.1080/07053436.2022.2140978.

[bibr57-10126902261418739] Quidu M Favier-Ambrosini B and Delalandre M (2025) Becoming a minimalist athlete: A sociological investigation into the forms, meanings, and lived experiences of minimalism within recreational sports. International Review for the Sociology of Sport: 1–22. DOI: 10.1177/10126902251344629.

[bibr58-10126902261418739] Rail G (2016) La violence de l’impératif du bien-être: Bio-autres, missions de sauvetage et justice sociale. STAPS 112(2): 17–31.

[bibr59-10126902261418739] Rapp A Tirabeni L (2018) Personal informatics for sport: Meaning, body, and social relations in amateur and elite athletes. ACM Transactions on Computer-Human Interaction 25(3): 1–30.

[bibr60-10126902261418739] Rooksby J Rost M Morrison A , et al. (2014) Personal tracking as lived informatics. Proceedings of the SIGCHI Conference on Human Factors in Computing Systems: 1163–1172.

[bibr61-10126902261418739] Rosa H (2018) Une sociologie de la relation au monde. Paris: La Découverte.

[bibr305-10126902261418739] Shilling C (2017) Body pedagogics: Embodiment, cognition and cultural transmission. *Sociology* 51(6): 1205-1221.

[bibr306-10126902261418739] Smith B and McGannon (2018). Developing rigor in qualitative research: Problems and opportunities within sport and exercise psychology. *International Review of Sport and Exercise Psychology* 11(1): 101-121.

[bibr62-10126902261418739] Soulé B (2007) Observation participante ou participation observante? Usages et justifications de la notion de participation observante en sciences sociales. Recherches Qualitatives 27(1): 127–140.

[bibr63-10126902261418739] Sparkes AC (2009) Ethnography and the senses: Challenges and possibilities. Qualitative Research in Sport and Exercise 1(1): 21–35.

[bibr64-10126902261418739] Stoler AL (2022) Carnal knowledge and imperial power: Gender and morality in the making of race. In: Alber E and Thelen T (eds) Politics and Kinship. London: Routledge, 142–175.

[bibr65-10126902261418739] Strava Press (2025) *Strava releases annual Year in Sport trend*. https://press.strava.com/fr/articles/strava-releases-annual-year-in-sport-trend.

[bibr307-10126902261418739] Tholander J (2025) The body as its own best sensor - an autoethnographic study of the sensitivities of the body in long-distance running. In: *Proceedings of the 2025 CHI Conference on Human Factors in Computing Systems (CHI '25)*. New York, NY, USA: Association for Computing Machinery, Article 122, 1-16. DOI: 10.1145/3706598.3713607.

[bibr66-10126902261418739] Toner J Allen-Collinson J Jackman PC , et al. (2023) I like to run to feel’: Embodiment and wearable mobile tracking devices in distance running. Qualitative Research in Sport, Exercise and Health 15(6): 805–818.

[bibr67-10126902261418739] Toner J Allen-Collinson J Jackman PC , et al. (2024) Wearables, wayfinding, and data visualisations for distance running. Performance Enhancement & Health 12(1): 100315.

[bibr68-10126902261418739] Wacquant L (2010) Participant observation/observant participation. In: Giddens A Sutton PW (eds) Sociology: Introductory Readings. Cambridge Malden: Polity Press, 69–72.

[bibr69-10126902261418739] Wacquant L (2015) Pour une sociologie de chair et de sang. Terrains & Travaux 26(1): 239–256.

[bibr70-10126902261418739] Wacquant L Vandebroeck D (2024) Carnal concepts in action: The diagonal sociology of loïc wacquant. Thesis Eleven 180(1): 111–143.

[bibr71-10126902261418739] Williams K (2015) An anxious alliance. In: Proceedings of the Fifth Decennial Aarhus Conference on Critical Alternatives, pp.121–131.

